# Endoplasmic Reticulum Stress, Oxidative Stress, and Rheumatic Diseases

**DOI:** 10.3390/antiox11071306

**Published:** 2022-06-29

**Authors:** Bruna Miglioranza Scavuzzi, Joseph Holoshitz

**Affiliations:** Department of Internal Medicine, University of Michigan, Ann Arbor, MI 48109, USA; bmiglior@med.umich.edu

**Keywords:** rheumatic diseases, endoplasmic reticulum stress, ER stress inhibit, oxidative stress, antioxidants

## Abstract

Background: The endoplasmic reticulum (ER) is a multi-functional organelle responsible for cellular homeostasis, protein synthesis, folding and secretion. It has been increasingly recognized that the loss of ER homeostasis plays a central role in the development of autoimmune inflammatory disorders, such as rheumatic diseases. Purpose/Main contents: Here, we review current knowledge of the contribution of ER stress to the pathogenesis of rheumatic diseases, with a focus on rheumatoid arthritis (RA) and systemic lupus erythematosus (SLE). We also review the interplay between protein folding and formation of reactive oxygen species (ROS), where ER stress induces oxidative stress (OS), which further aggravates the accumulation of misfolded proteins and oxidation, in a vicious cycle. Intervention studies targeting ER stress and oxidative stress in the context of rheumatic diseases are also reviewed. Conclusions: Loss of ER homeostasis is a significant factor in the pathogeneses of RA and SLE. Targeting ER stress, unfolded protein response (UPR) pathways and oxidative stress in these diseases both in vitro and in animal models have shown promising results and deserve further investigation.

## 1. Introduction

Rheumatic and musculoskeletal diseases (RMDs) are a heterogenous group of inflammatory conditions that usually affect joints, muscles, bones, tendons and ligaments, but can also affect any other organ of the body [1]. There are over 200 known RMDs, which include, among other conditions, ankylosing spondylitis (AS), rheumatoid arthritis (RA), fibromyalgia, scleroderma, Sjögren’s syndrome and systemic lupus erythematosus (SLE). Many RMDs are progressive and impact quality of life and life expectancy [1].

Disease development in SLE is a result of a culmination of genetic, immunoregulatory, epigenetic, hormonal, and environmental influences. The disease is characterized by abnormal T cell signaling, B cell hyperactivity, production of autoantibodies, such as antinuclear (ANA) and anti-double-stranded DNA (dsDNA) antibodies, and formation of immune complexes (ICs), causing multisystem inflammation, and tissue and organ damage (e.g., lupus nephritis, LN) [2,3]. RA is a chronic inflammatory disease affecting primarily the synovial joints that can cause severe disabling symptoms. The disease is commonly characterized by increased synovial cell proliferation, inflammatory cell infiltration, damage in the lining of joints, and the presence of auto antibodies, such as the anti-citrullinated protein antibodies (ACPAs), a frequently observed marker in rheumatoid factor seropositive RA [4].

The endoplasmic reticulum (ER) is a multi-functional organelle best known for its role in protein folding [5]. When unfolded or misfolded proteins accumulate in the ER lumen, ER stress ensues [6]. To restore homeostasis, cells respond to ER stress with the activation of the unfolded protein response (UPR) [7]. Many studies have suggested a causal relationship between ER stress, persistent activation of the UPR and oxidative stress in the pathogeneses of RMDs and reported promising therapeutic effects upon the use of ER stress inhibitors and antioxidants [8,9,10,11,12,13,14]. Here, we review ER stress and oxidative stress and discuss relevant therapeutic targets in the context of two prototypic RMDs: SLE and RA.

## 2. The Endoplasmic Reticulum and ER Stress

The ER is responsible for synthesis and folding of approximately one third of the entire proteome in eukaryotic cells [5], it is also involved in several other processes such as protein quality control, protein transport, lipid biogenesis, intracellular calcium ion (Ca^2+^) storage, and autophagic vacuole formation [15,16]. Specialized molecular chaperones including 78-kDa glucose-regulated protein (GRP78) and 94-kDa GRP (GRP94), lectin chaperones (e.g., calnexin and calreticulin), and folding enzymes such as protein disulfide isomerases (PDIs) all act to prevent misfolding, aberrant interactions and aggregation of nascent proteins, thereby assuring conformationally intact and functionally active proteins [7,17]. However, even in physiologic conditions, a large fraction of newly synthesized proteins misfolds, and are degraded for clearance in a process named ER-associated degradation (ERAD), which involves recognition, targeting, ubiquitination, and retro-translocation of the misfolded proteins into the cytoplasm for proteasomal degradation [18,19]. In conditions where protein production increases or when there are cellular stressors, including alterations in redox status, ER calcium depletion, energy deprivation, deficient autophagy, and increased inflammation [6,20], the ER degradation capacity can be exceeded, leading to an accumulation of proteins in the ER lumen, which is known as ER stress [6]. Cells respond to ER stress with the activation of UPR [7].

ER stress has been implicated in the pathogenesis of several human leukocyte antigen (HLA)-associated disorders, including RA and AS [21,22,23]. HLA is a cluster of genes located on the short arm of chromosome 6 p21.3, which encode for cell surface glycoproteins, best known for their role in presentation of antigenic peptides [24,25]. HLA alleles and haplotypes are some of the most significant genetic risk factors in many human diseases [26], such as RA, in which the HLA-DRB1 locus, is the most significant susceptibility factor [4]. It has been long established that HLA-DRB1 alleles encoding a five-amino acid sequence motif called the “shared epitope” (SE) are carried by the majority of RA patients [27,28]. It has been recently demonstrated that upon induction of ER stress (with lipopolysaccharide or dithiothreitol), the SE receptor calreticulin, translocates from the ER to the cell surface, increases intracellular Ca^2+^ levels, activates peptidylarginine deiminase (PAD) enzyme, which in turn facilitates protein citrullination [23], which is central for the development of autoimmunity in RA [29]. In the same study, parenteral administration of lipopolysaccharide to transgenic mice carrying a SE-coding *DRB1* allele was found to lead to in vivo generation of ACPAs, increase serum levels of tumor necrosis factor alpha (TNF-α) and bone erosions [23].

Another case in point, certain HLA-B27 alleles are major genetic risk factors in AS [30]. One of the hypotheses to explain how HLA-B27 contributes to AS pathogenesis is based on the HLA-B27 tendency to misfold, which contributes to accumulation in the ER, increased ERAD, ER stress, and UPR activation [22]. In transgenic rats, the HLA-B27 misfolding and UPR were shown to be followed by an induction of the pro-inflammatory cytokine interleukin -23 (IL-23), indicating a link between HLA-B27 misfolding and immune dysregulation [31].

## 3. The Unfolded Protein Response

**Figure 1:**
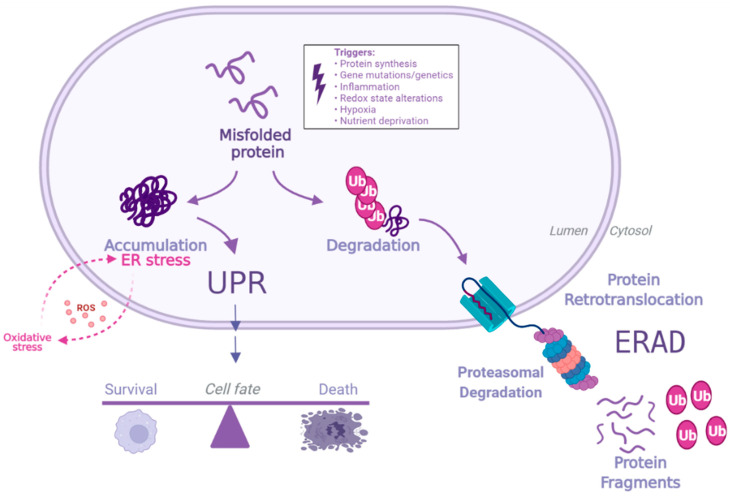
**Protein misfolding ER stress, and the UPR.** A large fraction of newly synthesized proteins commonly misfolds. Such proteins either associate with molecular chaperones to be remodeled into native proteins or can be degraded for clearance via ER-associated degradation (ERAD). When increased protein production occurs, or in the presence of cellular stressors, the ER degradation capacity can be exceeded, leading to an accumulation of proteins in the ER lumen, which causes ER stress. Such stress can induce oxidative stress, further contributing to accumulation of misfolded proteins and oxidation, creating a vicious cycle. ER stress is balanced by activation of the UPR, a process aimed at restoring homeostasis. However, if ER stress is prolonged or severe, the UPR can also promote cell death. Created with BioRender.com (accessed on 31 May 2022).

When ER stress occurs, UPR is induced to resolve accumulation of misfolded proteins and restore homeostasis [20,32]. The signaling events in UPR involve three master regulators: inositol-requiring kinase 1 (IRE1), protein kinase R-like endoplasmic reticulum kinase (PERK) and activating transcription factor 6 (ATF6). When in their inactive states, these transmembrane sensor proteins have their ER luminal domains bound to binding immunoglobulin protein (BiP), also known as GRP78 and Heat Shock Protein Family A Member 5 (HSP5A). However, when misfolded proteins accumulate, BiP is competitively released, by binding with higher affinity to misfolded proteins than the sensor proteins [7], thereby activating the proteins ATF6, IRE1 and PERK, which together orchestrate the adaptive signaling cascades known as the UPR. However, if the insult is prolonged or severe, the UPR can also promote cellular death [20], as schematically represented in Figure 1. Further, a growing body of research on the topic has revealed that the UPR participates in a wide range of crosstalk networks beyond resolution of protein load aberrations, such as innate immunity and cell differentiation [33]. Thus, ER stress and the UPR signaling are implicated in numerous pathological states including cancer, neurodegeneration, autoimmune conditions and RMDs [21,33,34].

Targeting protein aggregation and ER stress with the use of chemical chaperone 4-phenylbutyric acid (4-PBA) [35], a United States Food and Drug Administration (FDA) approved drug for treating chronic urea cycle disorders [36], has shown cytoprotective results in several conditions, including RMDs [37,38]. In a resiquimod-induced SLE mouse model, treatment with 4-PBA significantly mitigated ER stress signaling, attenuated splenomegaly—a common clinical manifestation in SLE -, reduced levels of TNF-α and anti-dsDNA, significantly ameliorated LN, reduced proportion of activated T and B lymphocytes and improved Treg-dependent immune suppression [39]. In another study involving a mouse model of SLE, treatment with 4-PBA increased renal expression of GRP78 and also mitigated renal injury development and progression [40]. These findings implicate noxious protein accumulation as a central component in the pathogenesis of SLE. The fact that inhibition of such accumulation can mitigate disease manifestations, suggests pharmaceutical targetability of the aberration.

Several other studies have targeted ER stress and specific UPR pathways in RA and SLE with encouraging results, indicating a pathogenic role for loss of ER homeostasis in these diseases. However, it is important to caution that although there is currently a wide range of molecules capable of modulating ER function in vitro and in animal models, substantial challenges remain in elucidating their precise mechanisms, as well as their specificities and safety. The currently available drug-like molecules aimed at targeting the UPR have been reviewed elsewhere [41].

### 3.1. IRE1 Signaling

When BiP is released, IRE1α oligomerizes and auto phosphorylates in trans, activates endoribonuclease and the splicing of X-box-binding protein 1 (*XBP1*) mRNA. Spliced XBP1 (*XPB1s*) translocates to the nucleus and upregulates genes involved in mitigating the protein burden, including those responsible for increasing protein folding capacity (e.g., BiP, other protein chaperones, PDIs), protein degradation (e.g., ERAD components, autophagy) and transport pathways [42,43,44]. In an additional process named regulated Ire1-dependent decay (RIDD), IRE1α promotes the degradation of mRNA substrates to reduce of protein-folding demand, thereby decreasing ER burden [45,46].

IRE1α-XBP1s axis activation has been implicated in the pathogenesis of RA, by the demonstration that XBP1 splicing induces toll-like receptor-dependent cytokine secretion, which in turn induces XBP1 splicing in synoviocytes, in a autocrine loop that sustains synovial fibroblasts activation [47]. In a study considering peripheral blood mononuclear cells (PBMCs) of RA patients compared to those of healthy controls, a significant increase in expression of *GRP78, IRE1,* and *XBP1s* was found for RA patients, while the unspliced *XBP1* form was dominant in healthy controls. The same study also demonstrated the involvement of RIDD targets, where (miRNA-17, -34a, -96, and -125b) were downregulated for RA patients [48]. An additional study evaluating the role of IRE1α pathway in RA in human patients and a mouse disease model, found a significant increase in IRE1α activation in macrophages from the synovial fluid of RA patients and that myeloid specific deletion of IRE1 in mice resulted in a protection against development of inflammatory arthritis. Additionally, and IRE1α inhibition with 8-formyl-7-hydroxy-4-methylcoumarin (4μ8C) (10 mg/kg per day, i.p.) suppressed joint inflammation in mice [49]. RA synovial fibroblasts (RASFs) have ‘tumor-like’ features, proliferating abnormally and these cells have been shown to have a reduced ability to undergo apoptosis [50,51,52]. STF-083010, an inhibitor of XBP1 splicing, was shown to reduce viability of primary cultured human RASFs, which are important pathophysiologic players in joint destruction in RA. In addition, the inhibitor suppressed synovial activity in an adjuvant-induced arthritis rat model [53]. Together, these findings underscore the contribution of hyperactive IRE1α/XBP1s axis to the pathogenesis of RA.

IRE-1 is a central regulator of B cell differentiation [54,55,56,57], and B cell hyperactivity is a defining pathogenic event in SLE [3,58], with B cell depletion therapies being considered for the treatment of the disorder [59]. A recent study evaluated the effect of STF-083010, in a pristane-induced lupus model [60]. In comparison to the pristane group, the pristane+ STF-083010 group showed attenuation in splenomegaly, as well as a significant decrease in XBP1s protein expression in the spleen. In blood samples, there was a decrease in mRNA expression of *Xbp1s*, while no modification in *Xbp1t* was observed. In B cells, it was observed that XBP1s-positive and CD19-positive B cells were significantly reduced in the pristane+ STF-083010 group in comparison to the pristane group. STF-083010 treatment suppressed pristane-induced B cell activation, plasma cell generation, immunoglobulin secretion, generation of B cell activating factors, and levels of TNF-α. No significant changes were observed in serum IL-6 levels among the groups. When considering levels of autoantibodies, STF-083010 treatment suppressed pristane-induced anti-dsDNA and anti-Smith antibody generation, with no differences observed among the three groups in relation to ANA. STF-083010 treatment also attenuated immunoglobulin deposition in the kidney and renal damage. No differential XBP1s expression was found in the kidneys among the three groups, suggesting that it is XBP1s activation in B cell, rather than in kidneys, that is driving renal damage [60].

Another recent study revealed that the IRE-1α/XBP-1 axis is upregulated in B cells of SLE patients, and correlates with stearoyl-coenzyme A desaturase (*Scd1* and *Scd2*) gene transcription and lipid accumulation in B cells of these patients, as compared to healthy controls [61]. In the same study, IRE-1α deficient mice showed improvement of survival, lower levels of proteinuria and glomerulonephritis severity, lower levels of anti-dsDNA antibodies and ANA, as well as lower levels of ICs deposition in kidneys as compared to littermate controls. *In vitro*, Ire1α deletion in B cells decreased cell survival and growth, and greatly impaired B cell differentiation into plasma cells [61]. Another group studied specific IRE-1α inhibitor, BI09 and found that it protected MRL.Fas^lpr^ mice against nephropathy, pulmonary and hepatic lymphocyte infiltration, autoreactive antibody formation, plasma cell differentiation and dramatically reduced B cell lipid volumes. Levels of autoimmune antibodies were restored to pre-treatment levels 4 weeks after interruption of treatment [61].

A role for IRE1α hyperactivity was also demonstrated in neutrophils from SLE patients and lupus prone MRL/lpr mice [62]. The authors reported enhanced XBP1 slicing in neutrophils isolated from patients with SLE as compared to the healthy controls. They also found correlations between the SLE disease activity score (SLEDAI) and the extent of XBP1 splicing. Furthermore, that study found that pretreatment of neutrophils with an IRE1α inhibitor, 4μ8C resulted in curtailed mitochondrial ROS (hydrogen peroxide) generation and inhibition of IC–mediated NETosis [62]. Together, these findings illustrate the importance of the UPR signaling in the pathogenesis of SLE and suggest a promising targeting potential for the IRE1α/XBP1s axis in the disease.

### 3.2. PERK Signaling

BiP release is associated with PERK dimerization and autophosphorylation. Once activated, PERK phosphorylates the eukaryotic initiation factor 2α (eIF2α), which can also be phosphorylated by PERK-independent mechanisms [63] and dramatically reduces the rate of most mRNA translation to alleviate burden of additional sources of ER stress, and favor cleaning mechanisms aimed at restoring homeostasis [64]. On the other hand, PERK activates *Atf4* mRNA to produce the activating transcription factor 4 (ATF4), involved in the activation of genes related to adaptation and relief of ER stress and oxidative stress [65]. ATF4 and PERK have been shown to upregulate expression of genes involved in resistance to oxidative stress; *Atf4*^−/−^ cells are sensitive to oxidative stress and have impaired expression of genes related to glutathione synthesis [66]. When ER stress is prolonged, ATF4 stimulates CCAAT-enhancer-binding protein homologous protein (CHOP) and other transcription factors involved in the initiation of programmed cell death [67,68]. Another PERK substrate is nuclear factor erythroid 2-related factor 2 (Nrf2), which translocates from the cytoplasm to the nucleus under stress conditions and regulates expression of antioxidant proteins, favoring cell survival during stress conditions [69,70]. Nrf2-/- cells have been shown to be prone to apoptosis and accumulation of ROS upon induction of the UPR [69]. PERK signaling plays an important role in maintaining bone homeostasis, and although more studies are necessary, there is a promising potential for the targeting of this pathway for bone diseases, which has recently been reviewed elsewhere [71].

A chemical compound named salubrinal is known to reduce ER stress by selective inhibiting of eIF2α dephosphorylation [72]. Salubrinal inhibition was shown to lower the release of proinflammatory cytokines (IL-1β, IL-2, IL-13 and TNF) as well as the expression levels of Dusp2, and to attenuate arthritis in an anti-collagen antibody mouse model of RA [73,74]. Salubrinal also ameliorated arthritis severity in collagen-induced arthritis (CIA) mice, manifested by lower clinical arthritis scores, synovium inflammation, joint damage, bone destruction, and the number of osteoclasts in the knee joints. Salubrinal inhibited in vitro osteoclast formation and suppressed RANKL-induced NF-kB signaling by promoting P65 degradation through the ubiquitin-proteasome system [75].

The role of the PERK-eIF2*α*-ATF4 axis in SLE has been underexplored, although impaired signaling in the PERK axis has been demonstrated in PBMCs from SLE patients, where downregulation of PERK was found, particularly in patient with severe condition (SLEDAI ≥ 12) [14]. Lower expression levels of IRE1, PERK, ATF6 and p-eIF2α were also found in T lymphocytes of SLE patients in comparison to healthy controls [76]. On the other hand, anti-dsDNA antibodies isolated from patients with LN were capable of binding to human mesangial cells and induce ER stress and NF-kB activation in these cells. In the same study, 4-PBA treatment downregulated the expression of IL-1β, TNF-α and MCP-1. Furthermore, ATF4 silencing with siRNA inhibited NF-kB activation and the levels of proinflammatory cytokines [77]. Another study evaluated the cause of increased frequencies of apoptosis in bone marrow mesenchymal stem cells of SLE patients; the authors found that in SLE patients, such stem cells showed ER stress, marked by increased protein expressions of p-PERK, pIRE-1, p-eIF2α and CHOP. As expected, a reduction of apoptosis and protein expression levels of CHOP and Jun N-terminal kinase1/2 (JNK1/2) was observed in these cells upon treatment with 4-PBA. In addition, PERK knockdown attenuated CHOP expression levels, and activated the anti-apoptotic regulator B-cell lymphoma-2 [78].

### 3.3. Activating Transcription Factor 6 Signaling

After BiP dissociation, ATF6 travels to the Golgi via coat protein complex II (COPII) vesicular transport, where in a process known as regulated intramembrane proteolysis (Rip), Golgi-localized proteases site-1 protease (S1P) and site-2 protease (S2P) cleave the cytoplasmic domain of ATF6, which then translocates to the nucleus, where it activates cytoprotective genes including those encoding the UPR, ERAD components, XBP1, and ER chaperone proteins (e.g., GRP78, GRP94, and calnexin) [79,80,81]. Under stress conditions, ATF6 has an important role in cell survival by activating genes that improve ER folding capacity, maintain ER healthy redox status, improve clearance of misfolded proteins, induce autophagy and mTor activation [82]. However, depending on conditions such as the intensity and duration of ER stress and ATF6 activation, it can also promote inflammation and cell death [82], given its known role in upregulation of CHOP and apoptosis [82,83,84,85].

There is a limited number of studies considering ATF6 in the context of RA. Although higher expression levels of ATF6 have been reported in RA synovium, and proinflammatory cytokines such as IL-1β and TNF have been shown to induced expression of *ATF6* in RAFLS [86,87]. Tacrolimus, an immunosuppressive drug used for the treatment of RA [88], has been shown to suppresses ER stress-mediated osteoclastogenesis and inflammation. In that study, tacrolimus was implicated in reducing several markers of ER stress in vitro and *in vivo*, including attenuation of ATF6 expression [14]. There have been a few studies evaluating the ATF6 pathway in the context of SLE [14,76,77], however, the role of this UPR axis in this disease has remained underexplored.

One of the hypotheses put forward to explain the link between HLA alleles and disease pathogenesis has been the major histocompatibility complex (MHC) cusp theory, which states that in addition to presenting antigens, “HLA molecules encode ligands in one of their hypervariable regions, designated a “cusp” based on its three-dimensional cusp-like conformation. Under certain environmental and background gene conditions, these cusp-ligands can interact with non- MHC receptors thereby activating aberrant cell signaling events that cause disease development” [89,90]. In a recent study considering the cusp theory, the transcriptional effects of three allelic epitopes in the HLA-DR cusp region (residues 65-79 of the DRβ chain) were explored in human (THP-1) and mouse (RAW 264.7) macrophages [91]. The authors found that the epitope encoded by the SLE-risk allele DRB1*03:01, the most significant genetic risk factor for SLE [92], activated a SLE transcriptome and triggered a cascade of SLE-associated cellular aberrations, including production of pro-inflammatory cytokines (IL-1β, IL-6, TNF-α), activation of proteasomal degradation and UPR pathways, reduction of intracellular ATP levels, loss of mitochondrial membrane potential, increase in mitochondrial superoxide production, and cell death by necroptosis; all of which were mitigated with 4-PBA treatment. In the same study, specific inhibitors of each UPR branch were evaluated; treatment with ceapin-A7, a specific ATF6α signaling blocker, significantly recovered intracellular ATP levels [91]. Furthermore, when stimulated by IFNγ ex vivo, bone marrow-derived macrophages from non-immunized transgenic (Tg) mice that carried the DRB1*03:01 allele presented activation of UPR and proteasomal degradation, reduction of intracellular ATP levels, as well as enhanced TNF-α and nitrite production, as compared to the Tg mice carrying the RA predisposing DRB1*04:01 allele and the RA-protective DRB1*04:02 alleles. Intraperitoneal injection of IFN-γ in the mice culminated in increased serum levels of anti-dsDNA, glomerular IC deposition and histopathological renal changes that resemble human LN [91]. These and other findings indicate a possible allele-specific contribution of ER stress to disease immune dysregulation [22,31,91].

A recent study in AS, demonstrated that ATF6 mediates fibroblast growth factor 2 transcription in chondrocytes, thereby aggravating angiogenesis and osteogenesis, processes which are central in the pathogenesis of the disease. In vivo, ATF6 inhibition with ceapin-A7 slowed the progression of osteogenesis by preventing angiogenesis-osteogenesis coupling [93].

**Figure 2:**
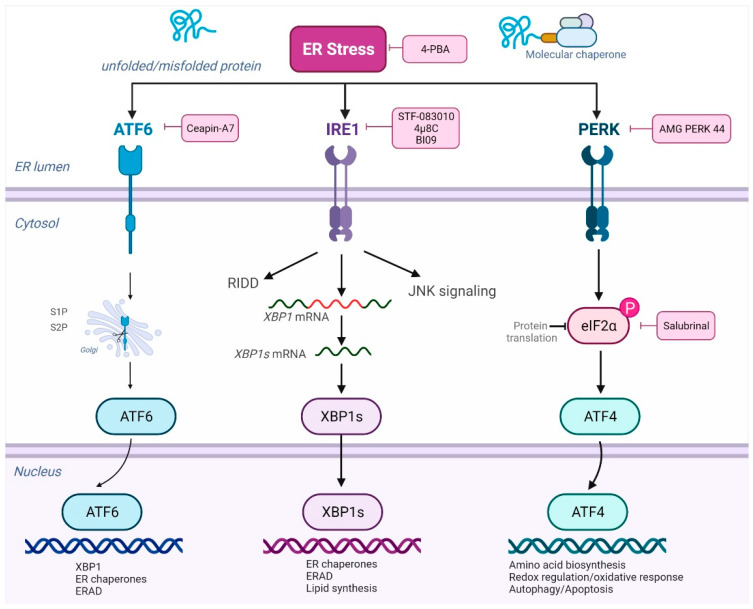
**ER stress, the UPR and pharmacological inhibitors.** ER stress can be prevented with the use of chemical chaperones, such as 4-PBA. Accumulation of proteins in the ER lumen activates the UPR, which involves three master regulators: ATF6, IRE1 and PERK, which have their ER luminal domains bound to BiP in their inactive domains. After BiP dissociation, ATF6 travels to the Golgi and proteases S1P and S2P cleave the cytoplasmic domain of ATF6, which then translocates to the nucleus, where it activates cytoprotective genes including those encoding ERAD components and ER chaperone. ATF6 signaling can be selectively inhibited with molecules such as ceapin-A7. When BiP is released, IRE1α activates endoribonuclease and the splicing of X-box-binding protein 1 (*XBP1*) mRNA. Spliced XBP1 (*XPB1s*) translocates to the nucleus and upregulates genes involved in mitigating the protein burden. IRE1 signaling can be inhibited with molecules such as 4μ8C, BI09 and STF-083010. Once activated, PERK phosphorylates eIF2α and activates *Atf4* mRNA to produce the activating transcription factor 4 (ATF4), involved in the activation of genes related to adaptation and relief of ER stress and oxidative stress. PERK signaling can be inhibited with molecules such as AMG PERK 44. Salubrinal can selectively inhibit eIF2α dephosphorylation. Created with BioRender.com (accessed on 31 May 2022).

In summary, several studies have targeted ER stress and specific UPR pathways in RA and SLE, indicating a pathogenic role for loss of ER homeostasis in these diseases. Figure 2 shows examples of such inhibitors and respective targets.

## 4. Molecular Chaperones

Molecular chaperones act to prevent aberrant protein aggregation by binding to unfolded and misfolded proteins [7,17]. In addition, they participate in biogenesis of MHC class I and class II molecules [94] and in antigen presentation [95], thereby having an important role in immunity. Given their conserved nature and abundance, particularly in stressed states, these molecules become favorable targets for regulatory T cells [96], and autoantibodies against molecular chaperones such as calnexin, GRP78 and GRP94 have been identified in SLE and RA, as well as numerous other rheumatic and inflammatory diseases [97,98].

GRP78 is a molecular chaperone and a key regulator of ER homeostasis, implicated in humoral and cellular autoimmune responses in RA, and a putative autoantigen in the disease [99,100,101]. Anti-GRP78 autoantibodies have been found in sera of as many as 63% of patients with RA, compared to 7% of patients with other RMDs and only 1% of healthy controls [102]. In another study, serum anti-GRP78 antibodies were found in 30% for RA patients compared to 10% of healthy control [100]. In addition to its antigenic properties, intraarticular injections of a selective GRP78 inducer activated synoviocyte proliferation and angiogenesis in the joints of mice with experimental osteoarthritis. Furthermore, in vitro experiments revealed that Grp78 small interfering RNA inhibited angiogenesis and synoviocyte proliferation [86]. It was also shown that the severity of CIA was significantly lower in Grp78+/− mice than in Grp78+/+ littermates [86], highlighting the potential role of this chaperone in the pathogenesis of the disease. Citrullinated GRP78 has also been recently shown to have a pathogenic role in RA [86,103,104].

Recently, it was demonstrated that azithromycin targets the UPR by inhibiting GRP78 activity, and that this treatment improved the severity of lesions comparably to the TNFα inhibitor etanercept in CIA mice. Additionally, deletion of the *GRP78* gene by CRISPR-Cas9 technique prevented anti-arthritic activity by azithromycin [105]. When evaluating T-cell proliferative response in peripheral blood and synovial fluid mononuclear cell preparations from RA participants, increased synovial proliferation in response to GRP78 was observed in 52% for RA patients compared to 17% of healthy controls [100]. The same group also found autoantibody reactivity to GRP78 in mice with CIA and pristane-induced arthritis. However, when GRP78 was administered to mice before collagen immunization, there was a prevention in the development of arthritis, suggesting an immunoregulatory role for GRP78 in arthritis, which has been supported by other studies [100,106,107,108].

Based on the previous observation that oral and nasal administration of specific peptides could induce Treg in animal models of autoimmune and inflammatory diseases [109], Shoda et al., 2015 studied two different HLA–DRB1*04:05-restricted epitopes of BiP (BiP ^336–355^ and BiP ^456–475^) and found they were differently recognized by effector and regulatory T cells [110]. While on the one hand BiP ^336–355^ induced PBMC proliferation and correlated with clinical arthritis activity and with the levels of circulating anti- BiP/citrullinated BiP antibodies, continuous oral administration of BiP ^456–475^ to mice with CIA resulted in improvement of joint inflammation and histologic scores, reduction of CD4+ T cell proliferation, increased numbers of CD4+CD25+FoxP3+ regulatory T cells and CD4+FoxP3+ T cells, and increased secretion of IL-10 from T cells. These findings illustrate the importance of the balance between effector and regulatory T cells in disease pathogenesis, as well as the immunomodulatory potential of BiP [108], highlighting its potential as an immunotherapeutic agent in RA [100,106,107,110].

A study evaluating T lymphocyte cell death in SLE patients revealed that upon treatment with thapsigargin, a known inducer of ER stress, T cells from SLE patients responded aberrantly, showing reductions in expression levels of GRP78, increased apoptosis, and reduced autophagic response in comparison to healthy controls [76]. Like RA, autoantibodies against GRP78 have been identified in SLE, although in lower titers and frequencies than in RA [97,104]. Levels of anti-GRP78 were found to correlate with brain barrier damages and the development of neuropsychiatric SLE [111]. Table 1 shows a summary of salient studies addressing ER stress, UPR pathways and chaperones in SLE and RA. It should be cautioned that while in vitro and in vivo studies show promising outcomes, many of these molecules have not yet been evaluated in translational and clinical studies, or in the context of RMDs.

## 5. Reactive Intermediates, Oxidative Stress, and the Interplay with ER Stress

ROS are reactive species generated endogenously or exogenously due to incomplete oxygen reduction, and include oxygen radicals (e.g., superoxide anion, hydroxyl radical, hydroperoxyl) and non-radical derivatives (e.g., hydrogen peroxide, H_2_O_2_) [112]. The main endogenous source of ROS are free radicals - particularly superoxide - arising from the mitochondrial respiratory chain. However, they can be generated from other sources including the ER, peroxisomes, macrophages, platelets, and leukocytes [10,113]. Exogenous triggers of ROS formation include ultraviolet radiation, certain drugs (e.g., acetaminophen), pollutants (e.g., cigarette smoking, pesticides), and bacterial or viral infections (e.g., Epstein-Barr) [10,114,115,116,117]. In controlled quantities, ROS modulate several physiological aspects of cell function and play roles in signaling pathways, such as those involved in T cell activation, cytokine production and proliferation, apoptosis of abnormal or aged cells, phagocytosis of infected cells, among other important physiological processes [118]. Nonetheless, when the production of ROS is excessive or when their elimination by antioxidant mechanisms is impaired, they may accumulate and become pathogenic, a condition known as oxidative stress [119,120]. Oxidative stress plays a central role in the development and exacerbation several chronic diseases, including SLE and RA [121,122].

Also relevant to autoimmunity and RMDs are reactive nitrogen species (RNS) (e.g., nitric oxide (NO) and peroxynitrite, produced mainly by mitochondrial nitrogen oxide synthetase (mtNOS) [112]. NO is a signaling molecule involved in numerous physiologic functions, including immune regulation, regulation of blood vessel tone, signal transduction (e.g., Ca^2+^ signaling) and regulation of apoptosis. However, when overproduced, and depending on the redox state of its cellular environment, NO may become harmful and react with ROS to form highly reactive molecules such as peroxynitrite, thereby generating new epitopes with the potential to break immune tolerance [123]. NO-induced tissue injury has been associated with a number of RMDs [124].

Epidemiologic, biologic, environmental, and genetic human studies, as well as animal models, have implicated oxidative stress in the pathogenesis of RA [125]. Consistent with that conclusion, impairments of antioxidative defense system [126], increased ROS formation, and oxidative damage to biomolecules have all been identified in serum and synovial fluids of RA patients [127,128] and in animal models [129]. Furthermore, biomarkers of oxidative stress have been found to correlate with higher disease activity [130], whereas interventions with products rich in antioxidants have produced disease amelioration [131,132,133].

Cigarette smoking, a major culprit for the development of RA, increases protein citrullination and oxidative stress [134,135], and has been shown to amplify the risk for RA development, in synergy with the number of SE-coding *HLA-DRB1* gene copies. For example, a 21-fold higher RA risk was found among patients who have had a smoking history and carried two SE-coding genes, compared to SE-negative nonsmokers [136].

Based on recent studies, the SE has been proposed as a signal transduction ligand [137] that interacts with cell surface calreticulin (CRT) [138,139] and initiates pro-oxidative [140,141] and pro-inflammatory signaling events [142,143] that facilitate bone damage and development of arthritis [125,144,145]. CRT surface expression can be triggered by ER stress and inflammation [23], favoring SE–CRT interactions. The interaction has been shown to increase intracellular Ca^2+^ levels, activate PAD, and increase protein citrullination [23,146,147], which, in turn serve as targets for autoantibodies [23,29]. Additionally, it has been demonstrated that aryl hydrocarbon receptor agonists (found in cigarette smoke) amplify SE- activated aberrant signaling, thereby augmenting the inflammatory response and bone erosive damage, and aggravate experimental arthritis in mice [147].

In SLE, the main source of ROS is overproduction by T-cell mitochondria due to mitochondrial hyperpolarization [148]. In this condition, the cytochromes within the electron transport chain are reduced, generating reactive species, such as the hydroxyl radical, the superoxide anion and hydroperoxyl [149]. Under physiological conditions, superoxide anion is converted to hydrogen peroxide through the action of superoxide dismutases, and can subsequently be converted to water through catalase [10]. However, when hydrogen peroxide is excessive and in contact with transition metals, e.g., ferrous ion, it can undergo Fenton reaction triggered by UV light, forming the hydroxyl radical, which cannot be neutralized. The hydroxyl radical causes modifications of cellular biomolecules such as lipids, proteins and nucleic acids (DNA and RNA) [10]. Oxidized biomolecules can generate new epitopes with immunogenic potential, which can cause the production of autoantibodies, bringing about inflammation, tissue damage, autoimmunity, and higher disease activity of SLE [8,9,150,151]. Targeting oxidative stress and mitochondrial oxidative stress has shown potential therapeutic benefits in several RMDs [152,153]. For example, idebenone (2,3-dimethoxy-5-methyl-6-(10-hydroxydecyl)-1,4-benzoquinonenoben), a synthetic analog of ubiquinone (Coenzyme Q10), modulates mitochondrial function and works as a potent antioxidant [154,155]. In MRL/lpr mice, idebenone treatment lowered mortality, disease activity and organ damage severity [156]. Another study evaluated the effect of trichloroethene (TCE) in MRL+/+ mice, with or without the use of acetylcysteine (NAC) - a precursor of the antioxidant glutathione. The study found that NAC supplementation significantly attenuated levels of TCE-induced ANA and 4-hydroxynonenal (HNE)-specific circulating ICs. Additionally, NAC supplementation inhibited TCE-induced inflammasome activation, B cell activation, NK cell infiltration in the liver, and histological changes. The authors hypothesized that NAC acted by blocking oxidative stress, which prevents neo-antigen formation and by blocking inflammasome activation [157].

The ER and mitochondria form structural and functional intraorganellar connections aimed at maintaining cellular homeostasis; the organelles cross-talk and coordinate processes such as redox signaling [158], Ca^2+^ transfer, cell death and inflammation [159,160,161]. These contact sites, known as Mitochondria Associated Membranes (MAMs), are rich in ER chaperones and have been described as a signaling hubs [162,163]. Calcium signaling plays a key role in ER-mitochondria communication and regulates multiple processes of cell metabolism, proliferation, differentiation, gene activation and cell death [164,165]. It has been demonstrated that when ER stress develops, the number of connections between the two organelles significantly increases, favoring Ca^2+^ uptake by the mitochondria and increase in production of ATP for the adaption to the condition of stress [162,166]. Conversely, alterations in MAMs functional properties have also been shown to favor ER stress and activate the UPR [162].

Since disulfide bond formation in the ER is a relevant source of ROS (H_2_O_2_), dysregulated disulfide bond formation/breakage can favor ROS accumulation and contribute to oxidative stress [66,167,168,169]. Furthermore, adequate protein folding is dependent on appropriate redox balance, increased oxidative stress can further impair the ER folding capacity and contribute to accumulation of misfolded proteins and oxidation, thereby aggravating the UPR response, ROS generation, inflammation, and trigger cell death [7,44,50]. Given the interplay between ER stress and ROS, ER stress inhibitors have been shown to reduce ROS. Conversely, antioxidants have been shown to reduce ER stress [170,171,172,173].

Examples of the role of ROS-ER stress interplay in RMDs include the reduction of mitochondrial hydrogen peroxide generation in neutrophils upon IRE1α inhibition with 4μ8C [62], 4-PBA-induced reduction of ROS generation in osteoarthritis tissues [174], reduction of mitochondrial superoxide with 4-PBA in human and mouse macrophages stimulated with the epitope encoded by SLE-risk allele DRB1*03:01 [91]. In addition, to evaluate if reduction of oxidative stress would influence inflammatory cytokine production and the expression of UPR genes in the context of AS, Navid et al., 2019 studied NAC - a precursor of the antioxidant glutathione - in bone marrow-derived macrophages from HLA-B27-transgenic rats, as compared to wild type control rats [175]. The study demonstrated a strong inhibition in the transcription of proinflammatory cytokines (IL-23, IL-12, Tnf, IL-6 and IL-1b), partial inhibition of UPR markers (e.g., Xbp1s, BiP and Chop) and alteration of metabolic activity in stimulated macrophages comparable to untreated controls [175].

## 6. Conclusions

Loss of ER homeostasis is a significant factor in the pathogeneses of RA and SLE, and while the elucidation of the mechanisms involved is still a work in progress, targeting ER stress, UPR pathways and oxidative stress in these diseases both in vitro and in animal models have shown promising results and deserve further investigation.

## Figures and Tables

**Table 1:** Summary of studies targeting endoplasmic reticulum stress in rheumatoid arthritis and systemic lupus erythematosus.

Ref.	System	Treatment	Outcome
[40]	Mouse model of SLE	4-PBA	↑ renal expression of BiP •mitigated the development and progression of renal injury
[77]	Human mesangial cells exposed to anti-dsDNA antibodies isolated from patients with LN	4-PBA	↓ expression of IL-1β, TNF-α and MCP-1
[78]	Bone marrow mesenchymal stem cells of SLE patients	4-PBA	↓ apoptosis ↓ protein expression levels of CHOP and JNK1/2
[91]	Human (THP-1) and mouse (RAW 264.7) macrophages activated with epitope encoded by SLE-risk allele DRB1*03:01 in presence of IFNγ	4-PBA	↓ activation of proteasomal degradation and UPR pathways •restored intracellular ATP levels •restored mitochondrial membrane potential ↓ mitochondrial ROS ↓ cell death
[49]	RA mouse model	4μ8C	↓ joint inflammation
[62]	Neutrophils from SLE patients	4μ8C	↓ mitochondrial ROS generation ↓ immune complex mediated NETosis
[53]	Primary cultured human RASFs; adjuvant-induced arthritis (AIA) rat model	STF-083010	↓ cell viability of primary cultured human RASFs ↓ synovial activity in AIA mouse model
[60]	Mouse model of SLE	STF-083010	Results for pristane+STF083010 group in comparison to the pristane group: •attenuated XBP1s expression in spleen and splenomegaly • mRNA expression of Xbp1s decreased • no effect on of Xbp1t in blood samples • Less XBP1s-positive and CD19-positive B cells • suppressed B cell activation, plasma cell generation, Ig secretion, generation of B cell activating factors, and levels of TNF-α • no significant changes in serum levels of IL-6 • suppressed dsDNA and anti-Smith antibody generation • no differences observed for ANAs • attenuated Ig deposition in the kidney and renal damage • no differential XBP1s expression was found in the kidneys
[61]	Mouse model of SLE	BI09	• mitigated progression of nephropathy • ↓ lymphocyte infiltration in lungs and liver, levels of autoreactive antibody, plasma cell differentiation and B cell lipid volumes • no effect observed for skin inflammation • levels of autoimmune antibodies were restored after interruption of treatment
[75]	RA mouse model	Salubrinal	• ↓ clinical score for arthritis, synovium inflammation, joint damage, degree of bone destruction, and number of osteoclasts in the knee joints • inhibited osteoclast formation and suppresses RANKL-induced NF-kB signaling via P65 degradation
[100]	RA mouse model (CIA)	BiP	↓ development of arthritis
[106]	DBA/1, HLA-DR1+/+, or interleukin-4 (IL-4)-knockout mice at the onset of arthritis	BiP (SQ or IV)	•suppressed established CIA in HLA-DR1+/+ and DBA/1 mice ↓ serum levels of anti-collagen IgG antibodies ↑ Th2 cytokines (IL-4) in T cells ↑ production of CII-specific IL-5, IL-10, and IFNγ at the termination of the study •development of severe CIA was prevented by the intravenous transfer of BiP-specific cells at the time of CIA induction in HLA-DR1+/+ mice •BiP failed to ameliorate the development of CIA in IL-4-/-, HLA-DR1+/+ mice
[107]	SCID mice with RASM engraftment	BiP (IV)	↓ cellular infiltrate in RASM transplants ↓ circulating IL-6 ↓ tissue inflammation in the RASM explants •downregulation of all quantifiable features of inflammation, HLA-DR, CD86, IL-6 and TNF-α in RASM transplants
[108]	PBMCs from RA patients	BiP	•secretion of an anti-inflammatory profile of cytokines •early stimulation of production of TNF-α •induction of IL-10 •incubation of monocytes in the presence of BiP induced long lasting down-regulation of CD86 and HLA–DR expression
[110]	RA mouse model (CIA)	BiP^456–475^ (PO)	•improvements in course of joint inflammation and histologic scores ↓ CD4+ T cell proliferation ↑ CD4+CD25+FoxP3+ regulatory T cells ↑CD4+FoxP3+ T cells ↑ secretion of IL-10 from T cells

↑, increased; ↓, reduced; 4-PBA, 4-phenylbutyric acid; 4μ8C, 8-formyl-7-hydroxy-4-methylcoumarin; AIA, adjuvant-induced arthritis; ANA, antinuclear antibodies; BiP, binding immunoglobulin protein; CHOP, CCAAT-enhancer-binding protein homologous protein; CIA, collagen-induced arthritis; IL, interleukin; IV, intravenous; LN, lupus nephritis; MCP-1, monocyte chemoattractant protein-1; PBMC, peripheral blood mononuclear cells; PO, orally; RA, rheumatoid arthritis; RASFs, RA synovial fibroblasts; RASM, Rheumatoid arthritis synovial membrane; ROS, reactive oxygen species; SCID, Severe combined immunodeficient mice; SLE, systemic lupus erythematosus; SQ, subcutaneous; TNF-α, tumor necrosis factor alpha.
